# Understanding the relationship between cognition and death: a within cohort examination of cognitive measures and mortality

**DOI:** 10.1007/s10654-018-0439-z

**Published:** 2018-09-10

**Authors:** Shabina A. Hayat, Robert Luben, Nichola Dalzell, Stephanie Moore, Eef Hogervorst, Fiona E. Matthews, Nick Wareham, Carol Brayne, Kay-Tee Khaw

**Affiliations:** 1 Department of Public Health and Primary Care, Institute of Public Health, University of Cambridge, Cambridge, UK; 2 Applied Cognitive Research Group, Loughborough University, National Centre for Sports and Exercise Medicine, Loughborough, UK; 3 Faculty of Medicine, Institute of Health and Society, Newcastle University, Newcastle, UK; 4 MRC Biostatistics Unit, Cambridge Biomedical Campus, Cambridge Institute of Public Health, Cambridge, UK; 5 MRC Epidemiology Unit, University of Cambridge School of Clinical Medicine, Cambridge, UK

**Keywords:** Cognitive function, Mortality, Population, Epidemiology

## Abstract

Despite several studies demonstrating an independent and inverse association between cognition and mortality, the nature of this association still remains unclear. To examine the association of cognition and mortality after accounting for sociodemographic, health and lifestyle factors and to explore both test and population characteristics influencing this relationship. In a population based cohort of 8585 men and women aged 48–92 years, who had cognitive assessments in 2006–2011 and were followed up till 2016 for mortality, we examined the relationship between individual cognitive tests as well as a global cognition score to compare their ability in predicting mortality and whether these differed by population characteristics. Risk of death was estimated using Cox proportional hazard regression models including sociodemographic, lifestyle and health variables, and self-reported comorbidities, as covariates in the models. Poor cognitive performance (bottom quartile of combined cognition score) was associated with higher risk of mortality, Hazard Ratio = 1.32 (95% Confidence Interval 1.09, 1.60); individual cognitive tests varied in their mortality associations and also performed differently in middle-age and older age groups. Poor cognitive performance is independently associated with higher mortality. This association is observed for global cognition and for specific cognitive abilities. Associations vary depending on the cognitive test (and domain) as well as population characteristics, namely age and education.

**Electronic supplementary material:**

The online version of this article (10.1007/s10654-018-0439-z) contains supplementary material, which is available to authorized users.

## Introduction

Cognitive decline occurs along a continuum [1, 2], spanning what has been described as ‘normal ageing’, to the other end of the spectrum of cognitive impairment and dementia. Studies have shown increased risk of mortality with dementia [3–5] and cognitive impairment [6–8]. However, in an ageing population, understanding the nature of this relationship across the continuum may provide insight into the different trajectories of decline. Poor cognitive function or mild impairment, has also been shown to be independently associated with subsequent mortality [9–12], both when measured globally and by specific cognitive domain [12–14].

Definitions of poor cognition differ across studies, classification based on varying local population norms and cut-offs depending on the assessment tool and population they are used in [15], make cross-study comparisons difficult. It is important to investigate whether less severe cognitive dysfunction or poor cognition has a higher mortality risk, not only because it precedes cognitive impairment and dementia [1, 16, 17], but also because it is likely to affect more individuals than those with impaired cognition and dementia as defined using accepted criteria. Studies examining association of milder cognitive difficulties with impending death have shown to be inconsistent [6, 7, 18].

Associations between cognition and mortality have not only been seen in later life when individuals are by definition at closer proximity to death [19, 20], but also at mid-life [21] and with level of performance as measured in childhood [11, 22]. The literature relates to testing both globally and by domain, based on theoretical models that have been put forward for conceptualising cognitive function [11, 23]. A common factor termed ‘g’ or general intelligence factor has been postulated as underlying all cognitive functions accounting for much of the variance observed in individuals and has been shown to be associated with mortality [6, 11], as have the more specific cognitive domains [12, 13]. It is still debated as to what is the best way of assessing cognitive function. [22].

The relationship between cognition and mortality is complex despite being ubiquitous [24]. Better cognitive ability is said to be an indicator of a well-functioning body influenced by genetic as well as early and later life biological and environmental factors. This includes the integrity of the brain and the efficiency of information processing, which has been suggested to be more strongly related to mortality than other cognitive abilities [25, 26]. Even though many cohort studies have shown robust associations between cognition and mortality, there still remains ambiguity on understanding this relationship and as yet no pathway or mechanism has been postulated.

Cross study comparisons are difficult due to differences in methodologies used. These include: inconsistencies in accounting for covariates that are associated with both cognitive function and mortality [10, 13, 27, 28]; using different cognitive tests; the use of selected groups, such as older individuals [29–32] or clinical patients [24], both of which are more likely to have co-existing morbidities. This has resulted in studies reporting different associations with mortality [13, 14, 21, 33]. The earlier hypotheses of terminal cognitive effects being greater in middle age and younger old and diminishing in later life have been refuted [10] and shown to continue to exist into oldest age, studies examining these age related differences in community dwelling older individuals have been limited.

The purpose of this study is to examine the association between cognitive performance (both global and domain specific) and mortality in a well characterised and relatively healthy population in mid to later life, to provide further clarity to this complex relationship. Our main aim is to investigate how specific cognitive abilities differ in predicting mortality and compare to a global cognition score after controlling for a range of known sociodemographic, health and lifestyle factors. In addition we examine the influence of the characteristics of the population tested, namely age and education.

## Methods

### Study participants and data collection

The European prospective investigation of cancer (EPIC) is a European wide study of diet and disease of which EPIC-Norfolk is one collaborating centre. At the inception of the study (1993–1997), EPIC-Norfolk recruited over 25,000 community-dwelling men and women (40–79 years old) from GP registers in and around the city of Norwich (Norfolk, United Kingdom). This involved the completion of a health and lifestyle questionnaire and a clinical examination [34]. Subsequent follow-ups have involved self-report of health and lifestyle postal questionnaires and further clinical assessments. The data presented here are from the third health examination (3HC or EPIC-Norfolk 3) which was conducted between 2006 and 2011 with a preceding pilot phase between 2004 and 2006, in participants aged 48–92 years, without any report of overt cognitive problems. The full assessment was a comprehensive 3-hour examination which included tests assessing different domains of cognitive function. A detailed description of the cohort both at inception and at 3HC have been published [35, 36]. The study was approved by the Norfolk Local Research Ethics Committee (05/Q0101/191) and East Norfolk and Waveney NHS Research Governance Committee (2005EC07L). Informed consent was obtained from all individual participants included in the study.

### Assessment of cognition

The EPIC-Norfolk cognition battery consisted of seven tests to assess performance across different cognitive domains. This battery has been described in detail previously [36]. The battery consists of: a shortened version of the Extended Mental State Exam (SF-EMSE) [37], assessing global function; the Hopkins Verbal Learning Test (HVLT, immediate total recall of three trials), assessing verbal episodic memory [38]; Cambridoge Neuropsychological Test Automated Battery Paired Associates Learning Test (CANTAB-PAL) [39, 40], using the first trial memory score (PAL-FTMS) as a measure of non-verbal episodic memory; a letter cancellation task assessing attention [41], using the accuracy score (PW-Accuracy); an event and time based task, for prospective memory [42]; the Visual Sensitivity Test (VST), with two separate outcome variables, VST-simple and VST-Complex [43, 44] for simple and complex visual processing speed (measured reaction time, in ms) and a shortened version of the National Adult Reading Test [45] or Short-NART [46], using the NART Error Score for measure of reading ability and crystallised intelligence [47]. This gave a total of eight different cognitive measures.

### Covariates

Weight was measured to the nearest 0.1 kg (using digital scales, Tanita) and height was measured with a stadiometer (Chasmores, UK) to the nearest 0.1 cm to calculate body mass index [BMI: weight (in kgs) divided by height (in m^2^)]. Education (the highest level attained) and social class were obtained from the baseline questionnaire. Education was categorised into three groups (1) No qualification (not completing school up to the age of 16), (2) Completion of school up to the age of 16 or up to the age of 18 and finally (3) those obtaining an education to graduate level (those who obtained a degree or equivalent) or above. Social class was dichotomised, into ‘non manual’ and ‘manual’ class. Self-report of smoking status (current, former or never smoker) and alcohol intake (Units/Week) were obtained from health and lifestyle questionnaire administered at the time of the clinic appointment. Alcohol units were categorised into 3 groups: 0 Units, 1–14 Units and more than 14 Units. Physical activity was assessed using two questions referring to activity during the past year, also from the baseline questionnaire. The first question asked about usual physical activity at work and the second question asked about the amount of time spent, in hours per week, in winter and summer in other physical activity. From this information, a 4 point index was derived using this information to categorise level of activity into (1) inactive (sedentary job and no recreational activity); (2) moderately inactive (sedentary job with, 0.5 hour recreational activity per day, or standing job with no recreational activity); (3) moderately active (sedentary job with 0.5–1 hour recreational activity per day, or standing job with, 0.5 hour recreational activity per day, or physical job with no recreational activity); and (4) active (sedentary job with 0.1 hour recreational activity per day, or standing job with 0.1 hour recreational activity per day, or physical job with at least some recreational activity, or heavy manual job). The validation of this index has been described in detail [48, 49].

Age was categorized into 5-year age bands. History of heart-attack, stroke, cancer, diabetes and depression were established using self-report of a range of conditions from health and life style follow up questionnaire.

### Mortality

Participants were followed up from the date of the cognitive examination until the date of their death or end of 31 March 2016, an average of 7.1 years. The cohort is linked to the NHS Central Register (NHS Digital) for health and the Office of National Statistics (UK) for death certification.

### Analyses

Measuring performance against the distribution of cognitive scores within a population to define abnormality, particularly where the data are not normally distributed has been described [15, 50]. The data for most of the tests in this study, were not normally distributed and the prevalence of dementia and cognitive impairment using accepted standard diagnostic criteria was very low in the cohort [35]. Preliminary examination across groups of approximate quartiles (due to the non-parametric distribution) did not show a linear relationship with mortality for all the cognitive tests (Supplementary Table S1). There seemed to be a more threshold response, with the lowest (approximate quartile) group having greater mortality than the other groups.

For this analysis, due to the distribution and non-linear response, associations were examined using approximate percentile cut-offs rather than the continuous cognitive score. Poor performance was defined as obtaining a score less than a cut-off point corresponding to approximately the 25th percentile of the population distribution in each of the eight cognitive measures individually. Participants were classified into two groups based on the cut-off scores for each of the tests. For prospective memory, poor performance was defined as those failing the task.

A composite score (EPIC-COGComp) was also created from the individual cognitive test, including the global measure of cognition, the SF-EMSE, which is an extension on the widely used Mini Mental state Exam (MMSE). The composite, should in theory, be a stronger measure of the cognition construct than any individual item, and here represents ‘g’ or general intelligence underlying all the cognitive functions assessed. Participants were classified in two groups for the continuous composite score in the same way as the scores were for the individual tests described above. A full description of how the composite score was created is given in “Appendix 1” (Supplementary Information) Briefly, for each of the individual cognition tests, a score of ‘0’ or ‘1’ was assigned based on whether the individual was in the ‘poor performance’ or ‘good performance or reference’ group for each of the eight cognitive outcome measures individually. The EPIC-COGComp was calculated as a sum of the score based on the performance group for all eight cognition test outcomes (range = 0–8). The approximate bottom quartile (or obtaining a score of 5 or below) for the composite score, was used to define poor performance for ‘g’.

The risk of death was estimated as a hazard ratio with 95 percent confidence interval (95% CI) for each of the cognitive tests in separate Cox proportional hazard regression models. The independent association of poor performance with mortality was assessed by first adjusting for age (per 5 years, treated as a continuous variable) and sex (models 1), then including education and social class (models 2) and finally extending the models to include other health variables (smoking, BMI, physical activity) and comorbidities (models 3).

Education, social class, physical activity and smoking were all treated as categorical variables in the analysis, as was co-morbidity (as present or not). Low and high BMI have stronger association with mortality than the intermediate groups (Table S2), however initial exploratory analyses showed little difference in hazard ratio when BMI was entered as a categorical (as low, normal, overweight and obese groups) or as a continuous variable (data not shown, but available at request). Therefore, BMI was entered in the model as a continuous variable to improve sensitivity of the analysis. The cognitive score was entered as a dichotomised variable based on the description above (poor performance or not). Including alcohol did not change the associations observed and so to reduce degrees of freedom and to increase stability of the models, we did not include alcohol in our final analysis.

In addition, we included the interaction terms 1/age group (≤ 65 and those > 65 years x each cognition test as the dichotomized variable) and 2/education group (Qualifications and No Qualifications × each cognition test as the dichotomized variable) to examine if age or education group contributed to performance for each test. Due to the strong influence of age and education on both cognition and mortality, the data were also stratified into age and education groups and adjusted hazard ratios calculated in each group. Stratification not only allows the examination of possible interaction, but examining the consistency of association in the different groups, permits the exploration of further potential confounding.

Spearman’s rank correlation coefficients were calculated using the continuous score for each of the individual tests, to examine the strength of relationship between each of the tests. The final analysis (model 4) mutually adjusted for all eight cognitive measures (entered as dichotomised variables as described above). Statistical analysis was performed using SPSS version 23.0 (IBM Corp., Armonk, NY, USA), with the level of significance set at 0.05.

### Missing data in cognitive tests

A number of sensitivity analyses were conducted to explore the effect of missing data. Hazard ratios were examined by assigning participants with missing data to either the poor performance or to the reference category. Hazard ratios also examined for individuals with data on all eight cognitive tests and the specified covariates (n = 5971) and compared to those with complete missing data of any of the eight cognitive measures as well as those not attending the health examination.

## Results

**Fig. 1:**
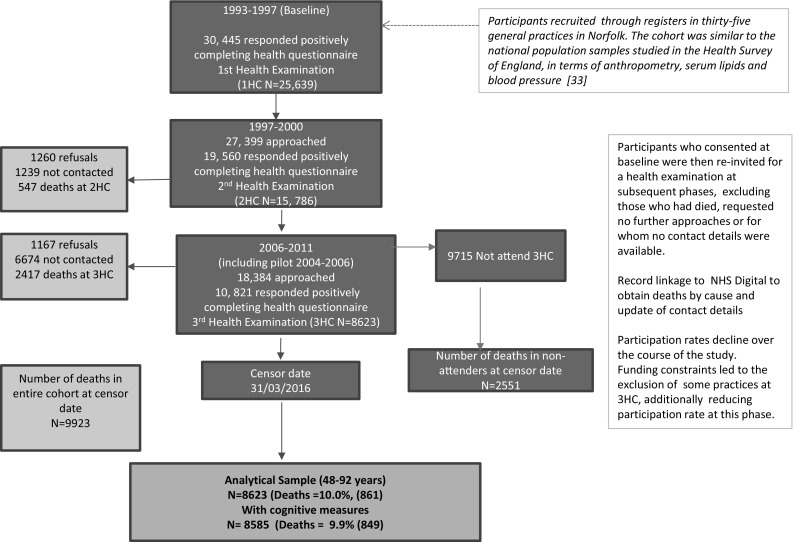
Selection of study participants in the EPIC-Norfolk 3 Study, (including pilot phase 2004–2006) for all-cause mortality, followed until 31 March 2016

After a maximum of 11.5 years of follow up (with an average of 7.1 years), there were 861 deaths in the 8623 participants taking part in EPIC-Norfolk 3. There were 849 deaths observed in the 8585 participants who had a cognitive tests measure (9.9% of the EPIC-Norfolk 3 cohort). Figure 1 summarises participation level at each phase and the selection of the analytical sample for this study.

Table 1 shows the means and proportions of the variables included in this analysis by survival status. There were significant differences between the two groups for almost all the variables examined. Those who died, were older, more likely to be men, have no qualifications, be physically inactive, to be non-drinkers, less likely to have been never smokers, and a higher proportion reported prevalent disease. Of the 8585 participants with cognitive data, 6128 participants had data for all the cognitive tests with 2457 having some of the test measures and 38 participants having none. These 38 participants were not included in the main analysis.

**Table 1:** Characteristics by survival status of 8585 participants with cognitive measures in the third health check phase of the European prospective investigation of cancer in Norfolk (EPIC-Norfolk 3) study, 2006–2011 (including pilot data, 2004–2006)

	Dead		Alive		*P* value
	N = 849		N = 7736		
Mean (SD)					
Age	75.6	(7.7)	67.9	(7.7)	< 0.001
Body mass index (kgs/m^2^)	27.1	(4.6)	26.8	(4.3)	0.08
Cognitive test score					
SF-EMSE	31.4	(4.1)	32.7	(3.0)	< 0.001
HVLT	22.6	(6.3)	25.3	(5.5)	< 0.001
PAL-FTMS	14.0	(4.8)	15.8	(4.2)	< 0.001
PW-accuracy	10.1	(6.4)	12.7	(6.0)	< 0.001
VST-simple (time, ms)	711.01	(211.3)	659.4	(160.7)	< 0.001
VST-complex (time, ms)	2320.9	(520.9)	2184.3	(417.03)	< 0.001
Sh-NART	17.4	(10.2)	17.2	(9.8)	0.5
Percent (N)					
Co-morbidity, % (n)					
Heart attack	9.9	(84)	2.7	(207)	< 0.001
Stroke	5.7	(48)	1.7	(134)	< 0.001
Cancer	15.8	(134)	8.7	(670)	< 0.001
Diabetes	5.7	(48)	2.7	(212)	< 0.001
Depression	11.5	(98)	8.3	(642)	0.001
Sex, men	57.4	(487)	43.4	(3354)	< 0.001
Education, no qualifications	33.5	(284)	25. 4	(1967)	< 0.001
Social class, manual	31.0	(261)	34.4	(2634)	0.05
Physical activity, inactive	56.8	(471)	35.1	(2678)	< 0.001
Smoking					
Current	5.5	(36)	4.3	(334)	< 0.001
Former	56.0	(366)	45.1	(3527)	
Never	38.4	(251)	50.6	(3952)	
Alcohol					
0 units	34.1	(275)	4.3	(328)	0.01
1–14 units/week	53.8	(343)	44.9	(3429)	
> 14 units/week	11.6	(94)	50.8	(3880)	
Cognitive test					
Pros. mem, failed	30.3	(246)	17.5	(1330)	< 0.001

Participants followed up until 31 March 2016

*P* values by *t* test or Chi sq for proportion

*HVLT* hopkins verbal learning test, *ms* milliseconds, *N* Number, *PAL-FTMS* paired associated learning, first trial memory score, *Pros. Mem* Prospective memory, *PW-Acc* PW-accuracy, *SD* standard deviation, *SF-EMSE* short form extended mental state exam, *Sh-NART* short national adult reading test, *VST* visual sensitivity test

Compared to those with incomplete or no data, those having attempted all the tests were younger, had higher average scores for all the tests, reported less co-morbidity, were less likely to be physically inactive, have no qualifications and be non-drinkers (Supplementary Table: S3). The age and sex adjusted hazard ratios for mortality for those who attended the health check and those who were invited but did not attend were examined. Using the group who had attended 3HC and had data on all 8 tests as reference, the mortality risk were as follows: with data on 1–7 tests, HR = 1.23 (95% CI 1.07, 1.41 *P* = 0.004); attended 3HC, but with no cog data HR = 1.71 (95% CI 0.96, 3.03 *P* = 0.07) and for those who were invited but did not attend 3HC, HR = 2.33 (95% CI 2.11, 2.56 *P* = < 0.001).

Table 2 shows the results of the Cox proportional hazards analyses for all the tests separately and for the composite score. For the age and sex adjusted models, there was an increased risk of mortality in those obtaining a poor performance score as compared to those who did not for each of the cognitive tests apart from the Short-NART. Additional adjustment for education and social class made little difference to the hazard ratios, as did the additional adjustment for co-variates (smoking, body mass index, physical activity) and comorbidities (models 3). Although the magnitude of the association varied slightly across the different tests, the PW-Accuracy test showed the strongest association, and was comparable to the association observed for the composite score.

**Table 2:** Poor performance as a predictor of mortality using the eight cognitive measures separately and a combined composite score as measured in the EPIC-Norfolk 3 Cohort (2006–2010), including pilot data (2004–2006) and followed up over average of 7.1 years, after adjustment for covariates

	Range^a^	N	Freq Mort.% (n)	Model 1			N	Model 2			n/N	Model 3		
				HR	95% CI	*P* value		HR	95% CI	*P* value		HR	95% CI	*P* value
SF-EMSE		8483					8402				795 /8273			
Good	32, 37	6178	8.0 (492)	1.00				1.00				1.00		
Poor	0, 31	2305	14.6 (337)	1.21	1.05, 1.39	0.01		1.22	1.05, 1.41	0.01		1.17	1.01, 1.36	0.05
			*P *< 0.001											
HVLT		8139					8063				735 /7944			
Good	22, 36	6102	7.5 (456)	1.00				1.00				1.00		
Poor	0, 21	2037	15.1(308)	1.21	1.04, 1.41	0.01		1.21	1.03, 1.41	0.02		1.19	1.01, 1.40	0.03
			*P *< 0.001											
PAL-FTMS		7461					7390				685 /7273			
Good	14, 26	5387	7.7 (416)	1.00				1.00				1.00		
Poor	0, 13	2074	14.3 (296)	1.16	1.00, 1.36	0.05		1.16	1.00, 1.36	0.06		1.18	1.01, 1.38	0.04
			*P *< 0.001											
PW-acc		8410					8331				778 /8205			
Good	10, 54	6073	7.5 (457)	1.00				1.00				1.00		
Poor	− 31, 9	2337	15.0 (351)	1.31	1.14, 1.52	*P *< 0.001		1.32	1.14, 1.53	*P *< 0.001		1.33	1.15, 1.54	*P *< 0.001
			*P *< 0.001											
		7144					7074				613 /6963			
VST-simple														
Good	453.1, 694.0	5356	7.7 (415)	1.00				1.00				1.00		
Poor	694.1, 4078.3	1788	12.4 (222)	1.25	1.06, 1.48	0.01		1.23	1.04, 1.46	0.02		1.22	1.03, 1.45	0.02
			*P *< 0.001											
VST-complex		7144					7074				613 / 6963			
Good	458.8, 7291.1	5358	7.3 (390)	1.00				1.00				1.00		
Poor	849.2, 11825.8	1786	13.8 (247)	1.28	1.08, 1.50	0.004		1.28	1.08, 1.51	0.003		1.26	1.07, 1.50	0.01
			*P *< 0.001											
Sh-NART		8112					8030				718 /7907			
Good	0, 24	6277	9.1 (573)	1.00				1.00	0.85, 1.29			1.00		
Poor	25, 50	1835	9.6 (176)	0.98	0.82, 1.16	0.8		0.97	0.81, 1.16	0.7		0.97	0.80, 1.17	0.7
			*P *= 0.6											
Pros. mem		8403					8324				784 /8199			
Success		6827	8.3 (567)	1.00				1.00				1.00		
Failure		1576	15.6 (246)	1.25	1.08, 1.46	0.004		1.25	1.07, 1.46	0.01		1.27	1.08, 1.49	0.003
			*P *< 0.001											
EPIC-COGComp^b^		6128					6067				504/ 5971			
Good	6, 8	4237	6.3 (267)	1.00				1.00				1.00		
Poor	0, 5	1891	13.5 (255)	1.27	1.06, 1.53	0.01		1.26	1.04, 1.52	0.02		1.31	1.09, 1.60	0.01
			*P *< 0.001											

Models 1 adjusted for age and sex; Models 2 adjusted for age, sex, education and social class; Models 3 adjusted for age, sex, education and social class, smoking, body mass index (bmi), physical activity and prevalent disease

*CI* confidence interval, *EPIC-COGComp* composite score (a summary score based on the performance group using data from all eight cognition test outcomes); 1, *Freq* frequency, *HVLT* hopkins verbal learning test, *Mort.* mortality, *N* number in the analyses, *n* number of deaths, *PAL-FTMS* paired associated learning, first trial memory score, *Pros. Mem* prospective memory, *PW-Acc* PW-accuracy, *SF-EMSE* short form extended mental state exam, *Sh-NART* short national adult reading test, *VST* visual sensitivity test

^a^Range of score (or in case of VST, reaction time in ms). ^b^The composite score is based on combining all the individual cognitive tests, so number in the final analysis (model 3) is significantly smaller than the other tests. See Supplementary information on the summary score

In the sensitivity analysis, imputing missing into the poor performance made little difference to the hazard ratios (with slightly strengthening associations for some), but attenuating considerably for most of the tests including the composite score when ‘missings’ were assigned to the reference category (Supplementary Table S4). Thus indicating that the ‘missings’ were likely to be in the poor performance group. Further sensitivity analyses, to compare those with measures on all eight tests, with those with seven tests or less, showed associations that were similar to those seen in the whole cohort analysis. Associations were statistically significant and stronger for participants with data on all eight tests, and considerably attenuated for those with data on seven tests or less. In the latter group, associations were observed for PW-Accuracy, VST complex and prospective memory, although not to significance. There was very little or no association for the remaining tests for those with incomplete data (Supplementary Table S5).

No significant interaction was observed with age group (≤ 65 vs. > 65 years) and any of the cognitive test (data not shown) and for education, only significant for HVLT (*P* = 0.03) but none of the other tests. On stratification, there seem to be some age group differences, with significant and stronger associations observed for the composite score, HVLT, PW-Accuracy and VST-Complex (Table 3) in the middle-age group. Weaker and mostly significant associations observed for composite and for all the other tests, except short-NART in the older age group. Stratifying by education group, associations with mortality were observed in the ‘no qualifications’ sub group for all tests apart from HVLT and weak but not significant for NART. Only weak (or no) association were observed in the ‘with qualifications’ sub group for all tests with strongest association observed for HVLT, PW-Accuracy and the composite score. (Table 4). The confidence intervals overlapped in both the age and education sub-groups.

**Table 3:** Association of poor performance and mortality, stratified by age group (equal to or younger than 65 years and over 65 years) in the eight cognitive measures separately and the combined composite cognition score

Test	Age <= 65 years				Age > 65 years			
	n/N	HR	(95% CI)	*P* value	n/N	HR	(95% CI)	*P* value
SF-EMSE	95/3102	1.19	(0.72, 1.97)	0.5	700/5171	1.17	(1.00, 1.37)	0.05
HVLT	96/3048	1.74	(1.05, 2.87)	0.03	639/4896	1.15	(0.97, 1.36)	0.1
PAL-FTMS	91/2843	1.15	(0.68, 1.92)	0.6	594/4430	1.18	(1.00, 1.39)	0.05
PW-accuracy	95/3088	1.60	(1.01, 2.54)	0.04	683/5117	1.29	(1.10, 1.50)	0.001
VST-simple	83/2683	1.11	(0.64, 1.92)	0.7	530/4280	1.25	(1.05, 1.50)	0.01
VST-complex	83/2683	1.68	(1.02, 2.75)	0.04	530/4280	1.23	(1.03, 1.47)	0.02
Sh-NART	90/3005	0.80	(0.46, 1.39)	0.4	628/4902	0.99	(0.81, 1.22)	0.9
Pros. mem	95/3086	1.27	(0.70, 2.30)	0.4	689/5113	1.26	(1.07, 1.49)	0.01
EPIC-COGComp	74/2383	1.76	(1.03, 3.02)	0.04	431/3590	1.28	(1.04, 1.56)	0.02

*CI* confidence interval, *HVLT* hopkins verbal learning test, *N* number included in the analysis, *n* number of deaths, *PAL-FTMS* paired associated learning, first trial memory score, *Pros. Mem* prospective memory, *PW-Acc* PW-accuracy, *SF-EMSE* short form extended mental state exam, *Sh-NART* short national adult reading test, *VST* visual sensitivity test

**Table 4:** Association of poor performance and mortality, stratified by education group (with Qualification and No Qualifications) in the eight cognitive measures separately and the combined composite cognition score

Test	With qualifications			*P* value	No qualifications			*P* value
	n/N	HR	(95% CI)		n/N	HR	95% CI	
SF-EMSE	526/6117	1.12	(0.92, 1.36)	0.2	269/2156	1.25	(0.97, 1.60)	0.08
HVLT	487/5896	1.33	(1.09, 1.62)	0.01	248/2048	0.99	(0.76, 1.28)	0.9
PAL-FTMS	450/5422	1.06	(0.87, 1.30)	0.6	235/1851	1.43	(1.10, 1.86)	0.01
PW-accuracy	517/6079	1.31	(1.09, 1.57)	0.004	261/2126	1.41	(1.09, 1.81)	0.01
VST-simple	411/5205	1.15	(0.93, 1.43)	0.2	202/1758	1.37	(1.03, 1.83)	0.03
VST-complex	411/5205	1.12	(0.91, 1.39)	0.3	202/1758	1.61	(1.20, 2.15)	0.001
Sh-NART	480/5879	0.84	(0.63, 1.11)	0.2	238/2028	1.14	(0.87, 1.49)	0.3
Prospective mem	521/6078	1.19	(0.97, 1.46)	0.1	263/2121	1.41	(1.09, 1.83)	0.01
EPIC-COGComp	342/4508	1.27	(1.00, 1.60)	0.05	162/1463	1.46	(1.03, 2.07)	0.04

The data were also tested for reverse causality, which is to examine whether the associations observed were as a result of those with disease pathology (and being closer to death) also having lower cognition. The analyses (model 3) were repeated for each of the tests individually and the composite score by excluding individuals who died within three years of follow-up after cognitive testing (N = 229). At population level, the results of the reduced sample did not show evidence of reverse causality with hazard ratios barely changing (results available on request).

Exclusions of deaths within three years of the cognitive test and then stratifying the data by age group showed a different result from the original stratified analyses. The hazard ratios in the older-age group showed little change. However the same was not observed for the middle-age group with variations in their prediction of mortality across the different tests. The association for most of the tests were attenuated (and due to small number, were no longer significant). The greatest (and significant) increase in association for the middle-age group was observed for HVLT HR = 2.19 (95% CI 1.20, 4.00). Association were also strengthened for prospective memory. The greatest differences observed across the two age-groups were also seen in HVLT and prospective memory (and remained, though to a lesser degree for composite score). The age group differences observed without exclusion of deaths, no longer remained for PW-Accuracy and VST-Complex.

Correlations between the different cognitive tests were weak to modest, with the strongest between the HVLT (verbal episodic memory) and SF-EMSE (global cognition), r = 0.48 and the weakest between the VST-Complex and Short-NART r = 0.06 (Table S7). Therefore, collinearity was not considered to be an issue when including all cognitive measures in the final model. The PW-Accuracy test remained the strongest independent predictor of mortality after mutually adjusting for all the other cognitive abilities (Table 5).

**Table 5:** Poor performance as a predictor of mortality using the eight cognitive measures separately and a combined composite score as measured in the EPIC-Norfolk 3 after adjusting for all co-variates and mutually adjusting for all other cognitive measures (model 4)

Test	N = 5971 (504 events)		
	HR	(95% CI)	*P* value
SF-EMSE	1.17	(0.95, 1.43)	0.1
HVLT	1.07	(0.87, 1.32)	0.5
PAL-FTMS	1.11	(0.91, 1.34)	0.3
PW-accuracy	1.27	(1.05, 1.54)	0.02
VST-simple	1.18	(0.97, 1.43)	0.1
VST-complex	1.19	(0.98, 1.44)	0.08
Sh-NART	0.90	(0.72, 1.13)	0.4
Pros. mem.	1.18	(0.96, 1.45)	0.1

*CI* confidence interval, *HVLT* hopkins verbal learning test, *N* number included in the analysis, *n* number of deaths, *PAL-FTMS* paired associated learning, first trial memory score, *Pros. Mem* prospective memory, *PW-Acc* PW-accuracy, *SF-EMSE* short form extended mental state exam, *Sh-NART* short national adult reading test, *VST* visual sensitivity test

## Discussion

This study presents a number of key findings. In this large prospective cohort study of relatively healthy individuals in mid to later life, poor cognitive performance was independently associated with higher mortality over an average of seven years of follow-up. Greater mortality was observed in the lowest (approximate quartile) group, showing the association to be a threshold effect similar to previous reports [50] rather than a gradient across the range of ability. Associations were not only observed for global cognitive function (using the composite score), but also for the individual tests covering a number of abilities or domains [36]. These associations remained after adjusting for sociodemographic, a range of lifestyle and health variables, including prevalent disease. Associations were not observed for the Short-NART. This was expected as accumulated knowledge is known to be more stable than other cognitive abilities until later life [22, 51].

Our study confirms the robust relationship between cognition and mortality [10, 12, 13, 20, 21] and that the ability to predict mortality not only exists for global cognition, but also across several cognitive domains [12, 13]. This is to varying degrees, with some specific abilities to be more powerful predictors than others. Population characteristics, particularly age and education also influenced the relationship and the predictive value of each test. Cognitive impairment, even at mild levels increases the risk of mortality [3]. Unlike previous reports of no association of mild impairment and mortality [52], our study has shown this relationship extends beyond to include poor performance, even before any evidence of impairment.

There are three possible explanations for the observed increased associations with mortality; (1) poor performance, which is on the trajectory of cognitive decline, is an early indicator of dementia, which reduces survival time; (2) cognition is not related, but the association is confounded by disease pathology (reverse causality) which is having a negative impact on cognition and increasing mortality; (3) poor cognition is having an indirect impact by those with lower cognition unable to engage in appropriate lifestyle and health behaviours, such as healthy diet, being physically active and not smoking. Also having poorer health literacy which may hinder the recognition of signs and symptoms of disease, seek medical attention and follow prescribed medication regimes. It is unclear which of the three possibilities could be in operation, it could be either or all three.

On initial analyses by age group, we found that associations between cognitive test performance and survival were stronger in individuals who were in the middle-age group than those who were over 65 years. However, this may be an artefact of a recognised methodological issue [10]. The majority of the survivors in middle-age group are expected to survive many years beyond the census date, whereas survivors in the older age group (being chronologically closer to death, be frailer, have more co-morbidities and disabilities) are more likely to die soon after the census date. Therefore, there are less differences between the cognitive scores of those who die shortly on either side of the census date in the older group, and the differences between deceased and survivors become more obscure. This incomplete investigation of the effects of survival duration in studies is a known restriction of the survival analysis methods [10].

We found little evidence of reverse causality at population level in our cohort when excluding individuals who died within three years of the health examination. Excluding these individuals also removed the differences initially observed by age group, confirming that associations are not restricted to middle age, but continue into older age [10]. However we did also observe the strengthening of the hazard ratios for HVLT and prospective memory and mortality in the middle-age group, not seen in the older age group. This indicates that dysfunction of memory (both episodic and prospective) is far more detrimental in terms of survival in middle age than it is in later life. These findings concur with those from the Whitehall Study that also showed memory to better predict risk of mortality in midlife [53].

The other observation to highlight is the variation in the VST measures across the age groups. This shows that the two measures may be assessing different abilities. The measures of VST-Simple may be a reflection of overall frailty, slowing of simple responses and indicative of accelerated physical ageing in the older age group, and is not as sensitive to normal cognitive ageing and in situations of reasonable motor speed. The reduced differential between the age groups for PW-Accuracy and VST-Complex, after excluding those who died within 3 years indicate the greater significance of processing speed in proximity to death than to chronological age. These functions are known to be affected by physiologic functioning strongly predict mortality [25].

Investigations in mortality by cause are required to examine these results in more detail. To examine the question of the of different pathologies, is beyond the scope of this paper, as this requires information on cause specific mortalities across the different domains assessed by the EPIC-Norfolk cognition battery. In the case of all-cause mortality, individual cognitive domains are generally comparable to the composite score though there are some individual variations [27].

With regards to education, being in the poor performance group (in general) had a greater disadvantage in terms of survival for the ‘no qualifications’ group than it did for the ‘with qualifications’ group. This was observed for the composite score and the individual tests (although not significant for SF-EMSE and NART). The association was not observed in the HVLT, a test of verbal episodic memory, that requires semantic knowledge [38]. Even though social class was adjusted for, it can be speculated, that education adds some advantage to survival that is beyond socio-economic status. Our results are line with previous findings [6], that better cognition does not give the survival advantage in circumstances of better socio-economic conditions as it does in lower socio-economic conditions. Having said that, the overall influence was seen in both education groups, providing further support of the independent relationship cognition and mortality.

In our population with no overt symptoms of cognitive impairment, we have shown that the relationship between cognition and mortality exists along the continuum to include poor cognitive performance and that this association is not restricted to the disease states of cognitive impairment and dementia. Although memory deficits are the most common precursors to dementia, prospective memory, processing speed and executive function have also been shown to be strong indicators of decline and mortality [54, 55]. This study adds further evidence to the importance of these measures as predictors of mortality in this relatively high functioning population.

Correlations between our cognitive tests were not high, suggesting that they measure different abilities. However, cognitive abilities do not work in isolation or independently of each other, with any given test making demands on a range of abilities. A single test cannot give a pure measure of a single cognitive ability [53], thus making it difficult to isolate the true contribution of the single measure being reported. Assessing cognition across domains provides detail to the size and nature of the relationship with mortality.

The limitation of this study is that of healthy volunteer bias and the decline in participation rate over the follow-up period as one would expect from an ageing cohort, with frailer individuals and those with lower function less likely to participate. By cutting out those at the lower end of the distribution, the cohort is likely to be healthier than those in the general population, and associations reported may be underestimated. We also observed a relatively large proportion of deaths in the middle age group occurred within 3 years. This may be a reflection of the health of the participants attending the health examination. The younger attendees attending the health examination, may have been available due to ill health stopping them from working or other activities, and older participants were the more able and fitter survivors able to attend the clinic. Both groups may therefore be slightly different from their counterparts in the general population. The mortality rate in the under 65 group was very small, and would need further numbers to see if the associations observed in this age group are robust.

Having highlighted the limitation of the participants in EPIC-Norfolk as healthier individuals, the cohort still includes a wide age range from mid to later life, is representative of both men and women and covers a broad range of socio-economic and education levels. Conducting this study in this healthier population has the advantage of less confounding from co-morbidities, a limitation in other studies of older or from selected clinical groups. These cognitive measures were part of a wider, comprehensive health examination (maximum length 3 hour). Those who were slower, less able and possibly less healthy individuals had less chance to complete all tests within the limited appointment time. This is further strengthened as associations were observed in healthier individuals with data on all 8 measures, but not in those completing fewer tests, also indicating that conducting this analysis in a less healthy cohort may not have shown similar associations. We have used various methods to deal with the issue of confounding, including stratification, multi-variate adjustment and excluding people who died within three years of the cognitive test and found associations between cognition and mortality to remain. Nevertheless, we cannot rule out residual confounding with other known and unknown risk factors may still be present.

Inconsistencies across studies may also be due to the heterogeneity in methodologies, in terms of assessment tools and the sample population. If tests purporting to measure the same ability are tapping into different cognitive and sensory abilities, they cannot be measuring the exact same construct. Adding to this complexity is the variation in the rate of decline across the abilities, each with different influence on performance and subsequently on the outcome measured.

One single test did not stand out as being the best predictor for mortality, however, we do not agree that individual cognitive domains are no better predictors than more general cognitive scores [27]. Using this argument for the sake of brevity is too simplistic. Our test for general cognition (SF-EMSE), testing a number of domains, did not perform as well as the composite score which was a combination of all the tests of the battery, or even some of the other tests measuring fewer abilities. By combining all tests and presenting as a single standardised score and not considering the separate abilities (as some studies have done), may result in missing on vital information that may then hinder interpretation.

Our findings support the conclusion that cognitive function is independently associated with death. However, we also emphasise the importance of giving due consideration to the characteristics of the sample population and psychometric properties of the assessment tools when interpreting results.

### Electronic supplementary material

Below is the link to the electronic supplementary material.

Supplementary material 1 (DOCX 60 kb)

## Appendix: Creating the cognition composite score from the EPIC-Norfolk cognition battery (EPIC-COGComp)

### Method

A composite score (EPIC-COGComp), in theory should be a stronger measure of overall cognition than any individual item. EPIC-COGComp represents all the abilities covered in the EPIC-Norfolk cognition battery. Briefly, for each of the individual cognition test (as listed in Table 6, with the predominant abilities assessed by each test), two groups were generated. These were based on the cut-off point corresponding to approximately the 25th percentile of the population distribution in each of the eight cognitive outcome measures individually; the ‘poor performance group’ and the ‘good performance or reference group’. Participants were then assigned a score of 0 if they were in the ‘poor performance’ group and a score of 1 if in the ‘good performance or reference’ group for each of the tests. The EPIC-COGComp composite score was then calculated as a sum of the score based on the performance group for all eight cognition test outcomes. The lowest possible score was 0 (being in the poor performance group for all 8 cognitive test outcomes) and the highest was 8, (being in the good performance group for all 8 cognitive test outcomes). The dichotomised variable of the EPIC-COGComp score was included in the analyses in the exact same way as the individual tests scores, with the approximate bottom quartile (or obtaining a score of 5 or below) defining poor performance for global cognition.

**Table 6:** List of the individual cognitive tests used in the EPIC-Norfolk 3

	Name and outcome measure	Predominant ability measured by test
1	A shortened version of the extended mental state exam (SF-EMSE)	Global function
2	Hopkins verbal learning test (HVLT)	Verbal episodic memory (immediate total recall of three trials
3	Cambridge neuropsychological test automated battery paired associates learning test	Non-verbal episodic memory
4	Letter cancellation task	Attention
5	Event and time based task	Prospective memory
	Visual sensitivity test (VST)	Simple and complex visual processing speed
6 7	(1) VST-simple (2) VST-complex	
8	Shortened version of the national adult reading test (Sh-NART)	Reading ability and crystallised intelligence

### Missing data

As the EPIC-COGComp, composite score relies on a score for all eight cognitive tests, it was only possible to examine associations with mortality for those with a score on all 8 outcomes, reducing the analysis to only 6128 participants. To maximise use of the data, two variants of the composite score were created to include all the available cognitive measures. The first variant (EPIC-COGComp0) was generated by imputing a score of 0 (assigning individuals to the ‘poor performance group’) for any test with a missing value and the second variant (EPIC-COGComp1) was generated by imputing a score of 1 (assigning individuals to the ‘good performance or reference group’) for any test with a missing value. The range for both variants were also 0–8.

### Analysis

The risk of death was estimated as a hazard ratio with 95 percent confidence interval (95% CI) for the composite score by Cox proportional hazard regression. The independent association of poor performance with mortality was assessed by first adjusting for age and sex (models 1), including education and social class (models 2) and finally extending the models to include other health variables (smoking, body mass index, physical activity) and comorbidities (models 3).
