# Supplementary material for: Understanding the relationship between cognition and death: a within cohort examination of cognitive measures and mortality
Source: Eur J Epidemiol. 2018 Sep 10;33(11):1049–62. doi: 10.1007/s10654-018-0439-z (PMC6208995; doi:10.1007/s10654-018-0439-z)
Supplement: Supplementary file 1 — Supplementary material 1 (DOCX 60 kb) [file 10654_2018_439_MOESM1_ESM.docx]

Table S1: Age and sex adjusted association with mortality across performance group (G, approximate quartile) for each cognitive tests for participants taking part in EPIC-Norfolk 3, 2006-2011 (including Data from the Pilot Phase 2004-2006)

|  |  |  | |  | Age and Sex adjusted | | | | |  |
| --- | --- | --- | --- | --- | --- | --- | --- | --- | --- | --- |
|  |  | Range of score/time | Deaths | | HR | (95% CI) | | P-Value | |  |
|  |  |  | | *Freq, % (N) |  |  | |  | |  |
| SF-EMSE | Freq, N |  | |  |  |  | |  | |  |
| G1 | 2305 | 0-31 | | 14.6 (337) | 1.23 | (0.92, 1.66) | | 0.2 | |  |
| G2 | 2276 | 32-33 | | 9.6 (219) | 1.03 | (0.76, 1.39) | | 0.9 | |  |
| G3 | 2898 | 34-35 | | 7.6 (219) | 1.02 | (0.75, 1.37) | | 0.9 | |  |
| G4 (Reference) | 1004 | 36-37 | | 5.4 (54) | 1.00 |  | |  | |  |
|  |  |  | | *P=<0.001 |  |  | |  | |  |
| HVLT |  |  | |  |  |  | |  | |  |
| G1 | 2037 | 0-22 | | 15.1 (308) | 1.22 | (0.97, 1.53) | | 0.09 | |  |
| G2 | 2514 | 23-26 | | 9.7 (243) | 1.11 | (0.88, 1.39) | | 0.4 | |  |
| G3 | 1640 | 27-29 | | 6.0 (99) | 0.80 | (0.61, 1.05) | | 0.1 | |  |
| G4 (Reference) | 1948 | 30-36 | | 5.9 (114) | 1.00 |  | |  | |  |
|  |  |  | | P=<0.001 |  |  | |  | |  |
| FTMS |  |  | |  |  |  | |  | |  |
| G1 | 2074 | 0-13 | | 14.3 (296) | 1.08 | (0.84, 1.41) | | 0.5 | |  |
| G2 | 2093 | 14-16 | | 9.2 (192) | 0.94 | (0.71, 1.22) | | 0.6 | |  |
| G3 | 2012 | 17-18 | | 7.4 (148) | 0.90 | (0.68, 1.19) | | 0.5 | |  |
| G4 (Reference) | 1282 | 19-26 | | 5.9 (76) | 1.00 |  | |  | |  |
|  |  |  | | P=<0.001 |  |  | |  | |  |
| PW_Acc |  |  | |  |  |  | |  | |  |
| G1 | 2337 | -31, 9 | | 15.0 (351) | 1.57 | (1.24, 1.97) | | <0.001 | |  |
| G2 | 1872 | 10-12 | | 9.9 (185) | 1.28 | (1.00, 1.63) | | 0.05 | |  |
| G3 | 2144 | 13-16 | | 8.0 (172) | 1.22 | (0.95, 1.57) | | 0.1 | |  |
| G4 (Reference) | 2057 | 17-54 | | 4.9 (100) | 1.00 |  | |  | |  |
|  |  |  | | P=<0.001 |  | |  | |  | |

Table S1 Continued.

|  |  |  |  | Age and Sex adjusted | | |
| --- | --- | --- | --- | --- | --- | --- |
|  |  | Range of score/time | Deaths (Freq) % (N) | HR | (95% CI) | P-Value |
|  |  |  |  |  |  |  |
| VST_Plain (time in ms) |  |  |  |  |  |  |
| G1 | 1788 | 694.11-4078.32 | 12.4 (222) | 1.30 | (1.03, 1.63) | 0.03 |
| G2 | 1786 | 623.79-694.00 | 9.5 (169) | 1.11 | (0.87, 1.40) | 0.4 |
| G3 | 1783 | 579.15-623.75 | 7.3 (131) | 0.99 | (0.77, 1.27) | 0.9 |
| G4 (Reference) | 1787 | 453.11-579.11 | 6.4 (115) | 1.00 |  |  |
|  |  |  | P=<0.001 |  |  |  |
| VST_Fuzzy (time in ms) |  |  |  |  |  |  |
| G1 | 1786 | 2387.29-11825.88 | 13.8 (247) | 1.30 | (1.04, 1.63) | 0.02 |
| G2 | 1786 | 2157.80-2387.16 | 8.3 (149) | 1.06 | (0.83, 1.35) | 0.6 |
| G3 | 1786 | 1955.37-2157.64 | 7.1 (126) | 0.99 | (0.77, 1.28) | 0.9 |
| G4 (Reference) | 1786 | 458.90-1955.31 | 6.4 (115) | 1.00 |  |  |
|  |  |  | P=<0.001 |  |  |  |
| Sh-NART |  |  |  |  |  |  |
| G1 | 1835 | 25-50 | 9.6 (176) | 0.91 | (0.74, 1.12) | 0.4 |
| G2 | 2219 | 16-24 | 9.2 (205) | 0.91 | (0.75, 1.11) | 0.3 |
| G3 | 2005 | 10-15 | 8.8 (177) | 0.91 | (0.74, 1.11) | 0.3 |
| G4 (Reference) | 2053 | 0-9 | 9.3 (191) | 1.00 |  |  |
|  |  |  |  |  |  |  |

*P values by T test or Chi sq for proportion

Abbreviations: CI, Confidence interval HVLT, Hopkins Verbal Learning Test, ms, milliseconds; : N, Number; PAL-FTMS, Paired Associated Learning, First Trial Memory Score; ; Pros. Mem, Prospective memory; PW-Acc, PW-Accuracy, SD, Standard deviation; SF-EMSE, Short Form Extended Mental State Exam; Sh-NART, Short National Adult Reading Test; VST, Visual Sensitivity Test

Table S2: Distribution of deaths by body mass index (BMI) category

|  |  | Deaths | Age and Sex adjusted | | |
| --- | --- | --- | --- | --- | --- |
|  | Freq | Freq, % (N) | HR | (95% CI) | P-Value |
| >20-25 kg/m2 (Normal) | 2854 | 8.8 (250) | 1.00 |  |  |
| <=20 kg/m2 (Low) | 216 | 15.7 (34) | 2.17 | (1.51, 3.11) | <0.001 |
| >25-30 kg/m2 (Overweight) | 3890 | 9.7 (379) | 0.94 | (0.80, 1.10) | 0.4 |
| >30 kg/m2 (Obese) | 1643 | 11.4 (187) | 1.32 | (1.09, 1.59) | 0.004 |
|  |  | *P=0.001 |  |  |  |

Table S3: Comparison of Characteristics of 6128 participants of the European Prospective Investigation of Cancer in Norfolk (EPIC-Norfolk 3) Study with all 8 cognitive measures and those with incomplete or no data.

|  | All 8 tests (N=6128) |  | 1-7 Tests (N=2457) |  | No Cog Data (N=38) | | P-Value |
| --- | --- | --- | --- | --- | --- | --- | --- |
| **Mean (SD)** |  |  |  |  |  |  |  |
| **Age** | 68.0 | (7.7) | 70.4 | (8.6) | 73.9 | (9.4) | <0.001 |
| **Body Mass Index (Kgs/M^2^)** | 26.8 | (4.3) | 26.9 | (4.4) | 27.4 | (4.7) | 0.5 |
| **Cognitive Test Score** |  |  |  |  |  |  |  |
| SF-EMSE | 32.9 | (2.7) | 32.0 | (4.0) | - |  | <0.001 |
| HVLT | 25.3 | (5.5) | 24.4 | (6.1) | - |  | <0.001 |
| PAL- FTMS | 15.8 | (4.2) | 14.9 | (4.7) | - |  | <0.001 |
| PW-Accuracy | 12.6 | (6.1) | 11.9 | (6.1) | - |  | <0.001 |
| VST-Simple | 660 .6 | (147.6) | 684.0 | (251.4) | - |  | <0.001 |
| VST-Complex | 2186.8 | (394.6) | 2255.0 | (592.7) | - |  | <0.001 |
| Sh-NART | 17.2 | (9.8) | 17.4 | (10.1) | - |  | 0.4 |
| Pros. Mem % failed (n) | 18.2 | (1117) | 20.2 | (459) | - |  | 0.04 |
| **Percent % (N)** |  |  |  |  |  |  |  |
| **Sex, men** | 44.8 | (2747) | 44.5 | (1094) | 52.6 | (20) | 0.6 |
| Education, no qualifications | 24.6 | (1510) | 30.2 | (741) | 47.4 | (18) | <0.001 |
| Social Class,  Manual | 33.5 | (2035) | 35.3 | (860) | 28.9 | (11) | 0.2 |
| Physical Activity, Inactive | 35.9 | (2168) | 40.4 | (981) | 60.0 | (21) | <0.001 |
| **Co-morbidity** |  |  |  |  |  |  |  |
| Heart attack | 3.0 | (186) | 4.3 | (105) | 5.3 | (2) | 0.01 |
| Stroke | 1.8 | (113) | 2.8 | (69) | 7.9 | (3) | 0.001 |
| Cancer | 9.1 | (559) | 10.0 | (245) | 10.5 | (4) | 0.5 |
| Diabetes | 2.9 | (179) | 3.3 | (81) | 5.3 | (2) | 0.5 |
| Depression | 21.8 | (1336) | 21.5 | (528) | 31.6 | (12) | 0.3 |
| **Mortality, Dead** | 6.7 | (410) | 10.6 | (261) | 31.6 | (12) | <0.001 |
| **Smoking, Current** | 4.4 | (263) | 4.4 | (107) | 5.7 | (2) | 0.9 |
| **Alcohol Intake**  **Units/week** |  |  |  |  |  |  |  |
| 0 | 28.1 | (1657) | 34.0 | (805) | 36.4 | (12) | <0.001 |
| 1-14 | 60.5 | (3566) | 54.6 | (1291) | 57.6 | (19) |  |
| >14 | 11.4 | (674) | 11.4 | (269) | 6.1 | (2) |  |

Hazard Ratios (Age and Sex adjusted)

| 8 Tests | | 1-7 Tests | | 0 Tests | | Non-Attenders | |
| --- | --- | --- | --- | --- | --- | --- | --- |
| HR |  | HR | 95% CI | HR | 95% CI | HR | 95% CI |
| 1.00 (Ref) | | 1.23 | (1.07. 1.41) | 1.71 | (0.96, 3.03) | 2.33 | (2.11, 2.56) |
|  |  | P= 0.004 | | P=0.07 | | P=<0.001 | |

P values by Anova, T test or Chi sq for proportion

Abbreviations: HVLT, Hopkins Verbal Learning Test, ms, milliseconds; : N, Number; PAL-FTMS, Paired Associated Learning, First Trial Memory Score; Pros. Mem, Prospective memory task; PW-Acc, PW-Accuracy, SD, Standard deviation; SF-EMSE, Short Form Extended Mental State Exam; Sh-NART, Short National Adult Reading Test; VST, Visual Sensitivity Test

Table S4: Sensitivity analysis to examine the impact of missing data on association of poor performance with mortality for each cognition score separately and the composite score after adjusting for age, sex, education and social class, smoking, body mass index (bmi), physical activity and prevalent disease. Hazard ratio for risk of death shown for (1) those with no missing values (2) missing values included as poor performers (3) missing values included in the reference (good) performers

| **Original** | | | | **Missing = Poor**  **n/N 811/8368** | | | | | | | | **Missing = Reference (Good)**  **n/N 811/8368** | | | | | | | | | | | | |  |  |
| --- | --- | --- | --- | --- | --- | --- | --- | --- | --- | --- | --- | --- | --- | --- | --- | --- | --- | --- | --- | --- | --- | --- | --- | --- | --- | --- |
|  | **n/N** | **HR** | | **95% CI** | **P-Value** | | | **HR** | | **95% CI** | | | **P-Value** | **HR** | | | **95% CI** | **P-Value** | | | | | | | |  |
| **SF-EMSE** | **795/8273** |  |  | | |  |  | |  | |  | | |  | |  | |  | | | | | | | | |
| Good |  | 1.00 | |  |  | | | 1.00 | |  | | |  | 1.00 | | |  |  | | | | | | | |  |
| Poor |  | 1.17 | | 1.01, 1.36 | 0.04 | | | 1.17 | | 1.01, 1.36 | | | 0.04 | 1.16 | 1.00, 1.35 | | | 0.05 | | | | | | | |  |
| **HVLT** | **735/7944** |  | |  |  | | |  | |  | | |  |  | | |  |  | | | | | | | |  |
| Good |  | 1.00 | |  |  | | | 1.00 | |  | | |  | 1.00 | | |  |  | | | | | | | |  |
| Poor |  | 1.19 | | 1.01, 1.40 | 0.03 | | | 1.20 | | 1.04, 1.39 | | | 0.01 | 1.13 | 0.97, 1.31 | | | 0.1 | | | | | | | |  |
| **PAL-FTMS** | **685/ 7273** |  | |  |  | | |  | |  | | |  |  | | |  |  | | | | | | | |  |
| Good |  | 1.00 | |  |  | | | 1.00 | |  | | |  | 1.00 | | |  |  | | | | | | | |  |
| Poor |  | 1.18 | | 1.01, 1.38 | 0.04 | | | 1.22 | | 1.05, 1.42 | | | 0.01 | 1.13 | 0.97, 1.32 | | | 0.1 | | | | | | | |  |
| **PW-Acc** | **778/ 8205** |  | |  |  | | |  | |  | | |  |  | | |  |  | | | | | | | |  |
| Good |  | 1.00 | |  |  | | | 1.00 | |  | | |  | 1.00 | | |  |  | | | | | | | |  |
| Poor |  | 1.33 | | 1.15, 1.54 | <0.001 | | | 1.34 | | 1.16, 1.54 | | | <0.001 | 1.30 | 1.12, 1.50 | | | <0.001 | | |  | | |  |  |  |
| **VST-Simple** | **613/6963** |  | |  |  | | |  | |  | | |  |  | | | | | | | | | |  |  |  |
| **Good** |  | 1.00 | |  |  | | | 1.00 | |  | | |  | 1.00 | | | | | | | | | |  |  |  |
| **Poor** |  | 1.23 | | 1.03, 1.45 | 0.02 | | | 1.31 | | 1.13, 1.51 | | | <0.001 | 1.12 0.96, 1.31 | | | | | 0.2 | | | |  |  |  |  |
| **VST-Complex** | **613/ 6963** |  | |  |  | | |  | |  | | |  |  |  | | | | | | | | | | | |
| **Good** |  | 1.00 | |  |  | | | 1.00 | |  | | |  | 1.00 |  | | | | | | | | | | | |
| **Poor** |  | 1.26 | | 1.07, 1.50 | 0.01 | | | 1.28 | | 1.12, 1.48 | | | 0.001 | 1.11 | 0.94, 1.30 | | | | | 0.2 | |  | | | | |

Table S4. Continued

| **Original** | | | | **Missing = Poor**  **n/N 811/8368** | | | | | | | | | **Missing = Reference (Good)**  **n/N 811/8368** | | | | | | |  |  |
| --- | --- | --- | --- | --- | --- | --- | --- | --- | --- | --- | --- | --- | --- | --- | --- | --- | --- | --- | --- | --- | --- |
|  | **n/N** | **HR** | | **95% CI** | | **P-Value** | | | **HR** | **95% CI** | | **P-Value** | | **HR** | | | **95% CI** | **P-Value** | | |  |
| **Sh NART** | **718/ 7907** |  |  | |  | |  |  | | |  | | |  | |  | | |  | | |
| Good |  | 1.00 | |  | |  | | | 1.00 |  | |  | | 1.00 | | |  |  | | |  |
| Poor |  | 0.98 | | 0.81, 1.18 | | 0.8 | | | 1.04 | 0.89, 1.22 | | 0.6 | | 0.95 | 0.79, 1.14 | | | 0.6 | | |  |
| **Pros. Mem** | **784/ 8199** |  | |  | |  | | |  |  | |  | |  | | |  |  | | |  |
| Good |  | 1.00 | |  | |  | | | 1.00 |  | |  | | 1.00 | | |  |  | | |  |
| Poor |  | 1.27 | | 1.08, 1.49 | | 0.003 | | | 1.23 | 1.06, 1.44 | | 0.01 | | 1.26 | 1.08, 1.48 | | | 0.004 | | |  |
| **EPIC- COGComp** | **504/5971** |  | |  | |  | | |  |  | |  | |  | | |  |  | | |  |
| Good |  | 1.00 | |  | |  | | | 1.00 |  | |  | | 1.00 | | |  |  | | |  |
| Poor |  | 1.32 | | 1.09, 1.60 | | 0.01 | | | 1.38 | 1.19, 1.61 | | <0.001 | | 1.16 | 1.00, 1.35 | | | 0.05 | | |  |

Abbreviations: CI, Confidence interval; EPIC- COGComp, Composite score (a summary score based on the performance group using data from all eight cognition test outcomes); 1; Freq, Frequency; HVLT, Hopkins Verbal Learning Test; Mort., Mortality; N, Number in the analyses, n, number of deaths; PAL-FTMS, Paired Associated Learning, First Trial Memory Score; PW-Acc, PW-Accuracy, SF-EMSE, Short Form Extended Mental State Exam; Sh-NART, Short National Adult Reading Test; VST, Visual Sensitivity Test

Table S5: Hazard ratios (95% CI), adjusted for age, sex, education and social class, smoking, body mass index (bmi), physical activity and prevalent disease by individual cognitive tests, for participants with all 8 cognitive measures and those with incomplete data (on 1-7 tests)

|  | **Data with all 8 tests (n=5971)** | | | **Data on 1-7 tests (N=Varies according to test)*** | | |
| --- | --- | --- | --- | --- | --- | --- |
|  | **HR** | **(95% CI)** | **P-Value** | **HR** | **(95% CI)** | **P-Value** |
| **SF-EMSE** |  |  |  |  |  |  |
| Good | 1.00 |  |  |  |  |  |
| Poor | 1.31 | (1.08, 1.58) | 0.01 | 0.98 | (0.76, 1.24) | 0.8 |
| **HVLT** |  |  |  |  |  |  |
| Good | 1.00 |  |  |  |  |  |
| Poor | 1.23 | (1.02, 1.50) | 0.04 | 1.06 | (0.79, 1.41) | 0.7 |
| **PAL FTMS** |  |  |  |  |  |  |
| Good | 1.00 |  |  |  |  |  |
| Poor | 1.24 | (1.03, 1.49) | 0.02 | 1.01 | (0.73, 1.35) | 0.9 |
| **PW-Acc** |  |  |  |  |  |  |
| Good | 1.00 |  |  |  |  |  |
| Poor | 1.37 | (1.14, 1.65) | 0.001 | 1.26 | (098, 1.62) | 0.07 |
| **VST-Simple** |  |  |  |  |  |  |
| Good | 1.00 |  |  |  |  |  |
| Poor | 1.31 | (1.08, 1.58) | 0.01 | 0.85 | (0.56, 1.29) | 0.4 |
| **VST-Complex** |  |  |  |  |  |  |
| Good | 1.00 |  |  |  |  |  |
| Poor | 1.29 | (1.07, 1.56) | 0.01 | 1.14 | (0.76, 1.70) | 0.5 |
| **Sh-NART** |  |  |  |  |  |  |
| Good | 1.00 |  |  |  |  |  |
| Poor | 1.01 | (0.81, 1.26) | 0.9 | 0.92 | (0.63, 1.34) | 0.7 |
| **Pros. Mem** | |  |  |  |  |  |
| Good | 1.00 |  |  |  |  |  |
| Poor | 1.31 | (1.07, 1.59) | 0.01 | 1.23 | (0.94, 1.61) | 0.1 |

*N Varies according to test

| SF-EMSE N= 2302 |
| --- |
| HVLT N= 1973 |
| PAL-FTMS N=1302 |
| PW_Acc N=2234 |
| VST= 992 |
| Sh-NART N=1936 |
| PMN= 2228 |

Abbreviations: HVLT, Hopkins Verbal Learning Test, ms, milliseconds; : N, Number; PAL-FTMS, Paired Associated Learning, First Trial Memory Score; PW-Acc, PW-Accuracy, Pros. Mem; Prospective memory task; SD, Standard deviation; SF-EMSE, Short Form Extended Mental State Exam; Sh-NART, Short National Adult Reading Test; VST, Visual Sensitivity Test

Table S6: Association between poor performance and mortality for eight cognitive measures separately (Model 3) stratified by age group (≤65 vs > 65 years) after excluding 229 individuals who died within 3 years of cognitive testing

|  | **Age <=65 Years** | | |  | | |  |  | **Age > 65 Years** | | |  |
| --- | --- | --- | --- | --- | --- | --- | --- | --- | --- | --- | --- | --- |
| **Test** | **n/N** | **HR** | **(95% CI)** | | **P-Value** | | **n/N** | | **HR** | **(95% CI)** | **P-**  **Value** | |
|  |  |  |  | |  | |  | |  |  |  | |
| **SF-EMSE** | 65/30722 | 0.89 | (0.47, 1.71) | | 0.5 | 521/4992 | | | 1.15 | (0.96, 1.38) | 0.1 | |
|  |  |  |  | |  |  | | |  |  |  | |
| **HVLT** | 65/3017 | 2.19 | (1.20, 4.00) | | 0.01 | 481/4992 | | | 1.22 | (1.01, 1.48) | 0.04 | |
|  |  |  |  | |  |  | | |  |  |  | |
| **PAL-FTMS** | 62/2814 | 1.05 | (0. 55, 2.01) | | 0.9 | 455/4291 | | | 1.27 | (1.05, 1.53) | 0.02 | |
|  |  |  |  | |  |  | | |  |  |  | |
| **PW-Accuracy** | 65/3058 | 1.29 | (0.72, 2.32) | | 0.4 | 509/ 4943 | | | 1.27 | (1.06, 1.52) | 0.01 | |
|  |  |  |  | |  |  | | |  |  |  | |
| **VST-Simple** | 52/2652 | 0.92 | (0.44, 1.91) | | 0.7 | 398/4148 | | | 1.30 | (1.06, 1.60) | 0.01 | |
|  |  |  |  | |  |  | | |  |  |  | |
| **VST-Complex** | 52/2652 | 1.30 | (0.66, 2.56) | | 0.5 | 398/4148 | | | 1.30 | (1.06, 1.59) | 0.01 | |
|  |  |  |  | |  |  | | |  |  |  | |
| **Sh- NART** | 59/2974 | 0.87 | (0.45, 1.71) | | 0.7 | 465/4739 | | | 0.87 | (0. 68, 1.11) | 0.3 | |
|  |  |  |  | |  |  | | |  |  |  | |
| **Pros. Mem** | 65/3056 | 1.63 | (0.84, 3.2) | | 0.2 | 512/4936 | | | 1.29 | (1.07, 1.57) | 0.01 | |
|  |  |  |  | |  |  | | |  |  |  | |
| **EPIC-COGComp** | 46/2355 | 1.55 | (0.76, 3.15) | | 0.2 | 331/3490 | | | 1.34 | (1.07, 1.69) | 0.02 | |

Abbreviations: CI, Confidence interval; HVLT, Hopkins Verbal Learning Test, N, Number included in the analysis; n, number of deaths, PAL-FTMS, Paired Associated Learning, First Trial Memory Score; ; Pros. Mem, Prospective memory; PW-Acc, PW-Accuracy, SF-EMSE, Short Form Extended Mental State Exam; Sh-NART, Short National Adult Reading Test; VST, Visual Sensitivity Test

Table S7: Spearman’s correlation coefficient between the eight separate cognitive measures in EPIC-Norfolk 3

|  | SF-EMSE | PW Accuracy | HVLT | PAL-FTMS | Pros. Mem | VST-Simple | VST-Complex |
| --- | --- | --- | --- | --- | --- | --- | --- |
| PW-Accuracy | 0.33** |  |  |  |  |  |  |
| HVLT | 0.48** | 0.32** |  |  |  |  |  |
| PAL-FTMS | 0.35** | 0.27** | 0.39** |  |  |  |  |
| Pros mem | 0.26** | 0.19** | 0.23** | 0.21** |  |  |  |
| VST-Simple | -0.20* | -0.17** | -0.17** | -0.16** | -0.08** |  |  |
| VST-Complex | -0.15** | -0.17** | -0.16** | -0.16** | -0.11** | 0.26** |  |
| Sh-NART | -0.38** | -0.21** | -0.34** | -0.21** | -0.13** | 0.08** | 0.06** |

Abbreviations: HVLT, Hopkins Verbal Learning Test, ms, milliseconds; : N, Number; PAL-FTMS, Paired Associated Learning, First Trial Memory Score; PW-Acc, PW-Accuracy, Pros. Mem; Prospective memory task; SD, Standard deviation; SF-EMSE, Short Form Extended Mental State Exam; Sh-NART, Short National Adult Reading Test; VST, Visual Sensitivity Test
